# Our Duty

**Published:** 1880-09

**Authors:** J. Allen Osmun

**Affiliations:** Newark, N. J.


ARTICLE V.

### OUR DUTY.

BY DR. J. ALLEN OSMUN, NEWARK, N. J.

Vigorous constitutions can be built up, or the foundations laid for them if the proper care is not neglected in the first years of childhood, And if there is anything that comes within the range of the Practitioner which calls forth his pity, and awakens his sympathy, it is when a little child is brought to him for the extraction of some temporary tooth or teeth; where the ruin is so complete that nothing remains but to use the forceps: and more so when he remembers that this suffering, and loss could have been so easily avoided that it is the direct result of some ones neglect and carelessness. Brethren "these things ought not so to be;" and we propose, in a brief way to see why it is thus, that parents are so careless; whether from ignorance or inherent stupidity.

Often when the parents are remonstrated with, and told how detrimental it is to the subsequent health of the mouth and body, that these teeth should be extracted so long before the work for which nature intended them is fulfilled, they often say: "why you dont fill the first teeth, do you?" or "I never heard of such a thing before."

This is ample proof that there has been some neglect and want of care in this direction. I often ask them—have you ever been to the dentist yourself for operation? and the answer is almost invariably in the affirmative. Then has your dentist never told you of the importance of saving the temporary teeth? And the answer is in the negative.

In trying to account for this, I can come to but one conclusion, that it must be carelessness on the part of the operator, or his not appreciating the importance of their functions. It may be that a fear of being misunderstood, and that the motives might be assigned to selfishness, deter him from offering advice; or when he has put the mouth under his care in thorough order, and given directions as to cleanliness, &c., he may think he has fulfilled his duty; but if so, he fails to grasp the whole domain of his calling.

Does not our duty reach out and take into consideration the care of young mouths? Does not our profession deal with the preventive as well as the curative? And if it is the duty of the dentist to attend to this important matter, the question naturally presents itself, "Does not this duty come within the province of the family physician?" Who has so much influence as he? On whose judgment is there so much reliance placed? And to whom do the parents look for advice and counsel in regard to the physical welfare of their household, as the family physician? And he being almost the only one who comes in contact with the "*little folks*," during the first years of their life, who can speak with intelligence and authority in this particular, we are of the opinion that it is his duty to advise and give counsel in this matter, for often when the dentist sees tne mouth for the first time, the ruin complete and beyond salvation.

If the physician has failed to give counsel in this direction, he certainly has not come up to the "higher life," which takes in everything which can benefit humanity. But I am sure it is because the family physician has so many other things, which he regards of more supreme importance, that this has been neglected, or may it not be he thinks it beneath him to notice such trifles as this appears on first inspection. I would remind all, to remember that "life is made up of seconds, and this is not a small matter, for often the seeds of disease are laid in childhood which in after years develope themselves into serious troubles.

We have yet to come to a realizing sense of the danger to our health that lies in *decaying teeth.* Decay is part of death. Atoms of decay planted in the tissues of our bodies are so many seeds of death, and as I have often seen many children with nearly every tooth decayed and foul, I have only to ask the thoughtful practitioner of either Medicine or Dentistry, what will be the result of such cases?

It is not nature's plan to shed the temporary teeth in this manner. She commences at the root and absorbs it so gradually, that the child never knows of it until some day it can be taken out by the fingers, the proper way for all temporary teeth to be lost, and in all perfectly healthy conditions we find it so. Our Heavenly Father must have intended that the temporary teeth should remain until the permanent teeth are ready to erupt, and if this is true, then to neglect them is not worthy of our professions.

It is not necessary to enter into the particulars of the evil results of premature extraction; of the many cases of irregularity where the anxiety to avoid, has been the *very means* of its being brought about; and so many cases of weak digestive organs caused by the improper mastication of food when young, for these cases will readily suggest themselves to the mind of the practitioner; how can a child masticate its food with comfort, if the molars are decayed or gone? The result in either case is pernicious.

Since God is right in sending some children into the world with rugged constitutions, and all with some elements of health and beauty, we are justified in doing all we can to aid nature and to make the personal appearance beautiful.

I have often spoken to physicians on this subject, and urged them to advise their patients to have their little ones mouths looked after, and I am surprised to see how little is known in this direction. They have apparently never given the subject any thought at all; but whenever it has been suggested to them they have at once seen the advantage of it. It is a subject that ought to receive more attention from both the Medical and Dental practitioner, and we look forward to the time when the physician and dentist will be in harmony with each other in everything that can benefit humanity in any way.

